# A Transformer-Based Model for Super-Resolution of Anime Image

**DOI:** 10.3390/s22218126

**Published:** 2022-10-24

**Authors:** Shizhuo Xu, Vibekananda Dutta, Xin He, Takafumi Matsumaru

**Affiliations:** 1 Graduate School of Information, Production and System, Waseda University, Kitakyushu 808-0135, Japan; 2 Institute of Micromechanics and Photonics, Faculty of Mechatronics, Warsaw University of Technology, 00-661 Warszawa, Poland

**Keywords:** super-resolution, anime image, swin transformer, image reconstruction, shallow feature, deep feature, low-frequency information, high-frequency information, anime dataset

## Abstract

Image super-resolution (ISR) technology aims to enhance resolution and improve image quality. It is widely applied to various real-world applications related to image processing, especially in medical images, while relatively little appliedto anime image production. Furthermore, contemporary ISR tools are often based on convolutional neural networks (CNNs), while few methods attempt to use transformers that perform well in other advanced vision tasks. We propose a so-called anime image super-resolution (AISR) method based on the Swin Transformer in this work. The work was carried out in several stages. First, a shallow feature extraction approach was employed to facilitate the features map of the input image’s low-frequency information, which mainly approximates the distribution of detailed information in a spatial structure (shallow feature). Next, we applied deep feature extraction to extract the image semantic information (deep feature). Finally, the image reconstruction method combines shallow and deep features to upsample the feature size and performs sub-pixel convolution to obtain many feature map channels. The novelty of the proposal is the enhancement of the low-frequency information using a Gaussian filter and the introduction of different window sizes to replace the patch merging operations in the Swin Transformer. A high-quality anime dataset was constructed to curb the effects of the model robustness on the online regime. We trained our model on this dataset and tested the model quality. We implement anime image super-resolution tasks at different magnifications (2×, 4×, 8×). The results were compared numerically and graphically with those delivered by conventional convolutional neural network-based and transformer-based methods. We demonstrate the experiments numerically using standard peak signal-to-noise ratio (PSNR) and structural similarity (SSIM), respectively. The series of experiments and ablation study showcase that our proposal outperforms others.

## 1. Introduction

### 1.1. Background

As the visual basis for human perception, images are essential for an individual to observe, express, and transmit information. Image resolution, as a set of essential parameters of images, is used to evaluate the richness of detailed information contained in the image. Image resolution reflects the ability of an image to delineate detailed information. High-resolution (HR) images typically contain greater pixel density, richer texture details, and higher reliability than low-resolution (LR) images. However, in practice, many images may be rendered in low resolution for various reasons (network bandwidth, memory limitations). Therefore, the requirement of reconstructing HR images from LR images through super-resolution techniques is significant. The strong demand for high-resolution images also exists in anime.

**Figure 1:**
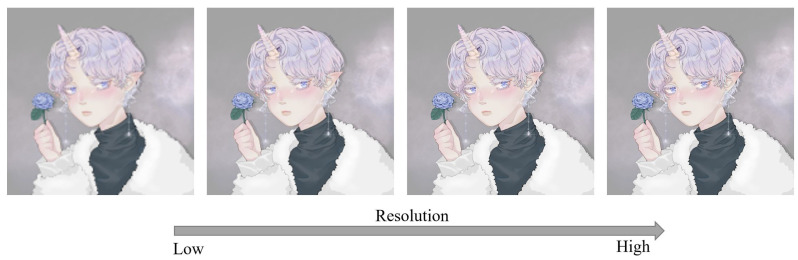
Anime image changes at different resolutions. The anime image resolution gradually increases from left to right. The image is adopted from [3]. We were given authorization to use an illustration copyrighted by Ms. Dai.

Anime combines graphic art, characterization, cinematography, and other forms of creative and individualistic techniques [1]. The earliest commercial anime in Japan dates back to 1917. A distinctive art style emerged in the 1960s with manga artist Osamu Tezuka’s work and spread over the following decades, developing a large domestic audience. With the popularization and promotion of Japanese anime, Japanese anime with various themes is respected and loved in Asia and worldwide. As of 2016, Japanese anime accounted for 60% of global animated TV shows [2]. In 2019, the annual export value of Japanese animation exceeded USD 10 billion. At the same time, with the improvement of image quality on various playback platforms, people have higher and higher requirements for the resolution of anime works. Anime works of different resolutions bring viewers different viewing experiences. As shown in Figure 1, even for the same anime image, we prefer the image with a higher resolution.

In addition to the influence of market requirements, anime creators and fans also have specific needs for the super-resolution technology of anime images. Super-resolution technology can help anime creators improve their works in higher resolution to meet audiences’ needs. Considering the needs of various parties, this work focuses on the field of anime image super-resolution and presents recent studies on image super-resolution techniques through literature surveys in the following section.

### 1.2. Image Super-Resolution Technology

#### 1.2.1. Fundamentals

Image resolution is the sum of the number of pixels in the image. Ten years ago, 320 px × 240 px resolution images were the mainstream in mobile phones, and their visual aesthetics were incomparable to the 2K resolution that can be witnessed everywhere in the digital world. Image super-resolution technology restores low-resolution images to high-resolution images, enhancing visual aesthetics.

Methods for image super-resolution reconstruction can be roughly divided into three categories: interpolation-based, refactoring-based, and learning-based methods. The method based on interpolation mainly calculates the relative distance between the new pixel and surrounding pixels and obtains the weight information according to the relative distance. Combined with the weight information, the exact value of the new pixel can be calculated. Common interpolation-based methods are nearest neighbor interpolation, bilinear interpolation, Bicubic interpolation [4], and so on. Refactoring-based methods are frequently used in image reconstruction, and common techniques include denoising, deblurring, and upsampling. The basic idea is to simulate the loss of image resolution in reality and find the factors that lead to the reduction of resolution through semaphore analysis, then use these factors to restore the detailed texture of the image. For example, in the iterative projection method [5], a high-resolution image is obtained by iterative backprojection by estimating the error between the low-resolution image and the generated image. In addition, statistical methods [6,7], patch-based methods [8,9], and sparse representation methods [10,11] are also used for image super-resolution tasks.

The idea of learning-based image super-resolution methods is to reconstruct low-resolution images into high-resolution images by learning the mapping relationship between low-resolution images and high-resolution images. Deep learning can adaptively learn the end-to-end mapping relationship through the network, and it has become the mainstream method in image processing. SRCNN [12] used the convolutional neural networks (CNNs) for the first time in the field of image super-resolution reconstruction and achieved some excellent results. Many CNN-based image super-resolution methods [13,14,15,16,17,18] since then have also demonstrated the effectiveness of CNNs in this task. SRGAN [19] is the first to introduce generative adversarial networks (GANs [20]) into image super-resolution tasks. SRGAN is a perceptual-driven method and pays more attention to the perception of the human eye. Since then, many GAN-based image super-resolution methods [21,22,23] have demonstrated theeffectiveness of GANs in this task.

Although the CNN-based and GAN-based methods efficiently perform the image super-resolution tasks, they usually need very deep networks to expand their receptive fields. Therefore, researchers have turned their attention to the transformer model [24]. TTSR [25] proposes a novel texture transformer network for image super-resolution, where low-resolution (LR) and reference (Ref) images are represented as query and key in the transformer, respectively. This is the first time that the idea of a transformer has been introduced into the field of image super-resolution. Since then, due to the outstanding performance of ViT [26] and Swin Transformer [27] in the field of the classification task, the two have also been introduced into the image super-resolution task [28,29].

#### 1.2.2. State-of-the-Art Techniques

(a) Common network structures

**Figure 2:**
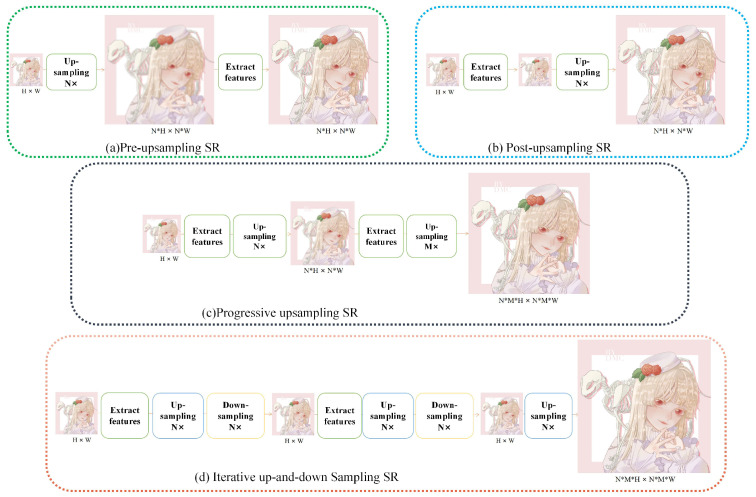
The network structure of super-resolution methods.

Starting from the early convolutional neural network (CNN)-based SRCNN [12], various deep learning methods have been applied to handle super-resolution tasks. The network structure of these deep learning methods can generally be roughly divided into four categories (Figure 2), namely, pre-upsampling SR, post-upsampling SR, progressive upsampling SR, and iterative up-and-down-sampling SR [30].

The pre-upsampling network model (see Figure 2a) first performs upsampling and then extracts deep features. It usually uses interpolation to implement upsampling operations to upscale the low-resolution image to the same size as the target image. The post-upsampling network (see Figure 2b) is the opposite of the pre-upsampling network. It places the learnable upsampling layer at the end of the network. A progressive upsampling network (see Figure 2c) can be seen as the splicing of multiple post-upsampling networks [31]. It performs this mainly to solve image super-resolution tasks at larger magnifications. The iterative up-and-down-sampling network (see Figure 2d) alternately builds up-and-down-sampling layers.

(b) Recent studies on image super-resolution reconstruction

A variety of learning-based methods have been applied to ISR reconstruction. In particular, there are three major categories: CNN-based, GAN-based, and recent promising SR methods using a transformer.

**CNN-based:** As for CNN-based methods, SRCNN [12] is the first one to use a convolutional neural network for ISR reconstruction. It uses a three-layer convolutional neural network to simulate the process of traditional image super-resolution reconstruction. Moreover, it achieves better results than traditional image super-resolution reconstruction. To improve its performance, there are many approaches, such as increasing network depth [13,15] and applying residual or dense blocks [14,16,17]. By using a very deep model consisting of 20 weight layers, VDSR [13] has better accuracy and visual effect. Moreover, the results show that deep networks with small convolution kernels can also obtain large receptive fields. EDSR [14] removes batch normalization layers, saving memory and speeding up computation. It has outstanding results and has become the reference model of many other methods [17]. DRRN [16] reduces the training difficulty of the model by introducing global residuals. In addition, a recursive recurrent unit is introduced, which can deepen the network without too much computation. ESPCN [18] proposes a new upsampling mode. The method proposed by ESPCN is a pixel rearrangement. It can extract feature information from the LR image at the beginning and then extend the LR feature information to the HR image through sub-pixel convolution.

**GAN-based:** Most of the previous CNN-based methods focus on maximizing the peak signal-to-noise ratio (PSNR), equivalent to minimizing the mean squared reconstruction error. Meanwhile, this also causes smoothing and blurring. Unlike human visual perception, the reconstructed images cannot meet our expectations. Therefore, SRGAN [19] introduces GAN [20] to the task of image super-resolution. ESRGAN [22] introduced the Residual-in-Residu Dense Block and used the VGG features before activation to improve the perceptual loss and achieved good results. Therefore, the PSNR of the output images is high, while texture details are still not acceptable. As for GAN-based methods, they can generate better images in visual effect. SRGAN is the pioneer that applies the GAN structure to tackle ISR tasks. It aims to modify the object function, adding the content loss function. Then, several variants have been proposed based on SRGAN.

**Transformer-based:** The two types of methods mentioned can only use local information of the image, and they ignore the global interaction among the parts of the image, resulting in low-quality restoration. The transformer is a new deep learning model that adopts the self-attention mechanism, differentially weighting the significance of each part of the input data. From the beginning, it has been a state-of-the-art method in natural language processing. Recently, due to its ability to tackle long-term dependency problems, the transformer has become increasingly popular in computer vision tasks, such as object detection, segmentation, classification, and ISR. It can exploit the input image’s local and global information, which will add a more detailed effect to the output image. This breakthrough attracts many scholars researching and introducing new network structures [28,29] for ISR.

### 1.3. The Research Questions, Rationale, and the Context for the Study

Methods based on CNN and GAN achieve better results than conventional interpolation methods. In order to obtain a larger receptive field, these networks leverage the deep network. This also introduces inconvenience to the training of the network. In addition, the actual receptive field size brought by deep networks is much smaller than the expected receptive field size. This is why transformers (ViT, Swin, etc.) have strong performance in downstream tasks because self-attention (the design form of query–key–value) is employed to calculate global information. Below, we mainly discuss methods based on CNN and GAN problems existing in the field of anime super-resolution. In addition, there is also the problem of limitations in current anime illustration datasets.

#### 1.3.1. Quality of Super-Resolution Images

**Figure 3:**
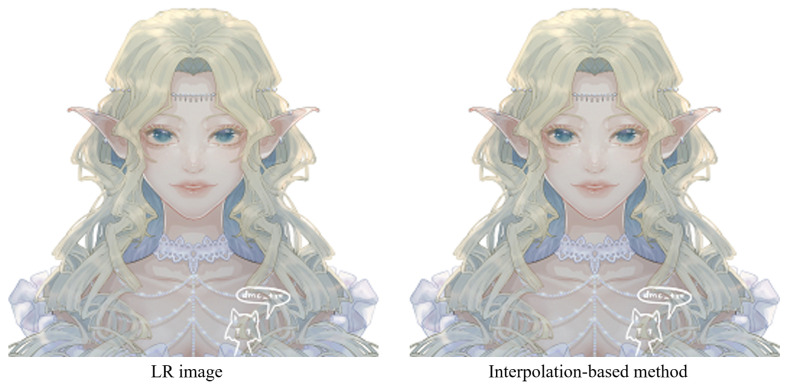
The blur result of interpolation-based algorithms. The image on the left is the original anime image. The image on the right is the super-resolution anime image obtained by the interpolation algorithm. The conventional interpolation algorithm does not consider the characteristics of the edge. After the image was processed, the blurring phenomenon can be noticed at the edge, which affects the quality of the image. The image is adopted from [3].

**(a) The interpolation-based algorithms will result in a serious blur effect when processing edges and textures.** The interpolation-based image super-resolution reconstruction algorithm is a relative method compared with others, and has low complexity. However, it is difficult to repair the high-frequency details of the image, leading to limited performance. There are some standard interpolation-based methods such as neighborhood interpolation, bilinear interpolation, and double legislative interpolation. They take the continuity of the image as the premise, assume that the gray value of the image is continuous, and consider that the gray value of the low-resolution image is the ideal sampling value. These methods will generate blurred images (see Figure 3) as they continue to determine the pixel block without saving the high-frequency information.

**(b) There are image blurs, checkerboard artifacts, hallucinations, and line misjudgments in deep-learning-based super-resolution algorithms.** The convolution kernel determines the receptive field of a convolutional neural network. The convolution kernel only pays attention to the local information of the image each time. Therefore, the interaction between the image and the convolution kernel is independent of the image’s content. This also makes it difficult for the convolution operation to obtain the global information of the image. Therefore, methods based on convolutional neural networks often suffer from image blurring and the appearance of checkerboard effects.

**Figure 4:**
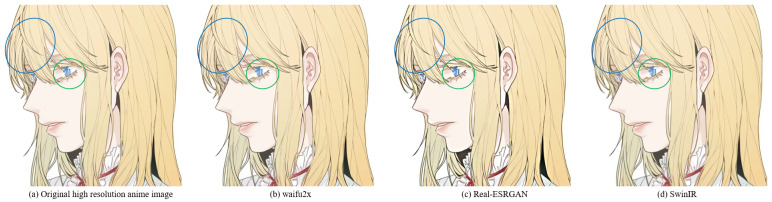
The results of super-resolution methods. (**a**) Original high-resolution anime image. (**b**) SR anime image generated by Waifu2x. (**c**) SR anime image generated by Real-ESRGAN. (**d**) SR anime image generated by SwinIR (image restoration using Swin Transformer). For Waifu2x, the blur and checkerboard artifacts problems are in the green and blue circles. For Real-ESRGAN, the circled area shows the hallucinations problem. For SwinIR, the circled area shows the lack of detail, such as line misjudgments. The image is adopted from [3].

For example, Waifu2x [32] is an anime super-resolution method based on convolutional neural networks. Subject to the shortcomings of the convolutional layer itself, the animation images generated by Waifu2x are often blurred (see Figure 4b). Although image super-resolution methods based on generative adversarial networks (GANs) have been proposed to alleviate the above problems, the resulting hallucinations also bring significant challenges to the SR task. For example, the Real-ESRGAN [23] model based on GAN achieves an excellent visual effect when performing anime image super-resolution tasks, but there are some hallucinations (see Figure 4c). The SwinIR based on the Swin Transformer improves the details of the generated image by enhancing the global perception ability; however, global awareness is reduced because it abandons the patch merging operation. Therefore, these lead to some lack of details in the generated anime images (see Figure 4d).

**(c) The ignoring of shallow features results in some missing details in the generated images.** The features extracted by the shallow network are relatively close to the input. It contains much low-frequency information. The low-frequency information of the image refers to the rough outline of the image, and the low-frequency information of low-resolution images and high-resolution images is similar. Unlike real-world images, there is not a lot of texture information in anime images. On the contrary, low-frequency information accounts for a large part. Therefore, the shallow features of anime images are of great significance for the super-resolution task of anime images.

#### 1.3.2. The Limitations of Anime Datasets

**(a) Lack of paired low-resolution and high-resolution anime images.** The research interest in super-resolution tasks of anime images is not very high. Paired datasets do not exist. In addition, some studies do not make the datasets they use open source. Therefore, we must make our own paired data to facilitate our research work.

**(b) Lack of high-quality anime data**. Three major factors affect the development of deep learning: algorithms, data, and computing power. All three are integral to any study. Data are the foundation, and any research is inseparable from data support. The corresponding research work cannot be carried out without a dataset. However, currently, there are few datasets in the field of anime image super-resolution. The most common one is Manga109 [33], but the number of samples it provides is only 109, and too few samples are not conducive to the training of the network model. Although the large-scale dataset Danbooru [34] provides larger-scale samples because the dataset is collected too randomly, a large number of pictures contain many wrong pictures. This also makes it unsuitable for our study. Meanwhile, in the case of limited computing power, using such a vast dataset for model training is unsuitable. Therefore, designing an anime dataset that meets our needs is necessary.

### 1.4. Contributions and Paper Outline

Due to the solid global perception of the capabilities of Swin Transformer, this work considers employing the advantages of Swin Transformer to the anime image super-resolution task. In addition, we propose targeted methods to solve the problems combined with the specific problems existing in the current anime super-resolution tasks. We state our contribution in this paper as follows:

- We proposed an anime image super-resolution network structure based on Swin Transformer.
- We modified the conventional Swin Transformer to improve the global awareness capability of the feature extraction network.
- We strengthened the extraction of low-frequency information given the richness of spatial information in anime images.
- Before the upsampling stage, shallow features were fused with deep features to provide more detailed information for the final result.
- The experimental results were compared numerically and graphically with those delivered by conventional convolutional neural network-based and transformer-based methods.
- The series of experiments and ablation study disclose anime image super-resolution tasks at different magnifications (2×, 4×, 8×).
- We constructed our anime dataset to compensate for the lack of anime super-resolution task datasets.
- Our approach speeds up the creative cycle for creators who can create at a low-resolution level and then revert to a high-resolution image.

This paper comprises five parts. Section 2 delineates the proposed method and presents how the image resolution task is achieved. The detailed description of the applied dataset is described in Section 3. Section 4 outlines the experimental results. Finally, Section 5 presents the conclusions and discusses the possibilities for future works.

## 2. Proposed Methods

**Figure 5:**
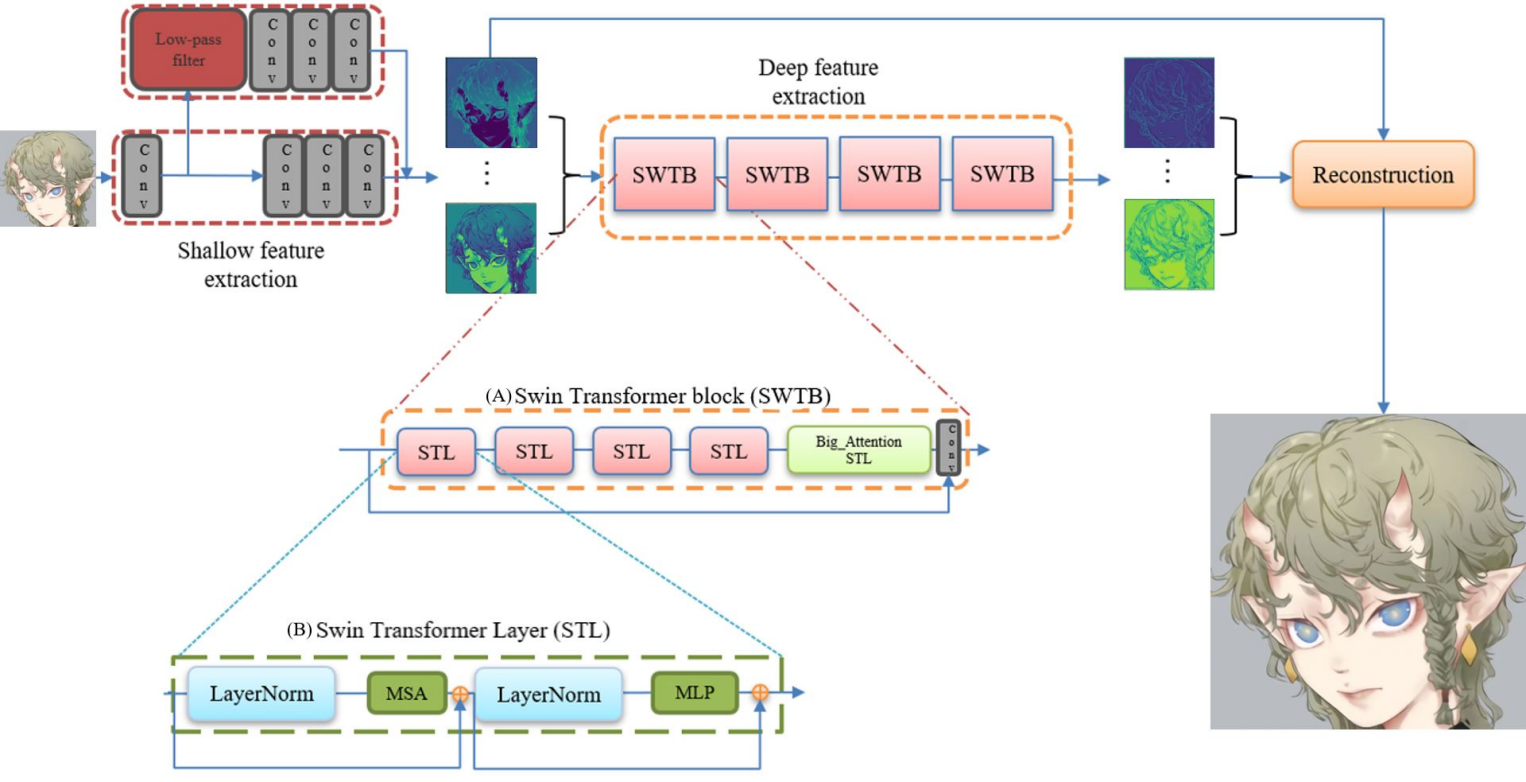
Overall network structure.

Our network model framework is mainly based on the post-upsampling SR framework. The network mainly consists of three parts (see Figure 5). The first is the shallow feature extraction part (based on CNN), the second part is the deep feature extraction network (based on Swin Transformer), and the third part is the image reconstruction network (based on PixelShuffle).

### 2.1. Shallow Feature Extraction Network

Shallow features are image features extracted by the shallow network (based on CNN). The receptive field of the shallow network is generally small. The overlapping area of the receptive field corresponding to each pixel of the feature map is also small, which ensures that the network can capture more detailed information in a spatial structure, for example, image color, texture, and edge information.

In super-resolution tasks, the majority of previous studies ignore the significance of shallow features, thus losing essential details. Shallow features extraction was introduced in SwinIR [28], SMIR [29], and RND [35] for image super-resolution tasks. SwinIR and SMIR use a convolutional layer to extract shallow features. Further, SFENet extracts shallow features with two convolutional layers in RDN. Therefore, this idea inspired this work and design of the corresponding shallow features for our specific anime image super-resolution task.

**Figure 6:**
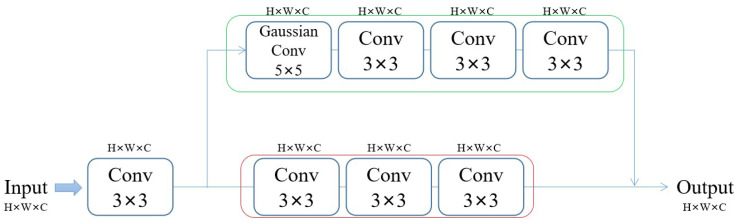
Shallow feature extraction network structure.

Most anime images consist of simple lines and textures compared to general drawings. Therefore, we should focus more on low-frequency information to extract the features of anime images. Thus, in addition to the normal feature extraction layer (red frame), we set a feature extraction layer for the low-frequency filtered data (green frame). Our shallow feature extraction network part is mainly based on a convolutional neural network (see Figure 6). Initially, the input image uses a convolutional layer with a 3 × 3 convolution kernel to extract features. These features are then passed into three 3 × 3 convolutional layers (red frame) and a Gaussian convolution kernel, followed by another three 3 × 3 convolutional layers to obtain image features that retain low-frequency information (green frame).

**Figure 7:**
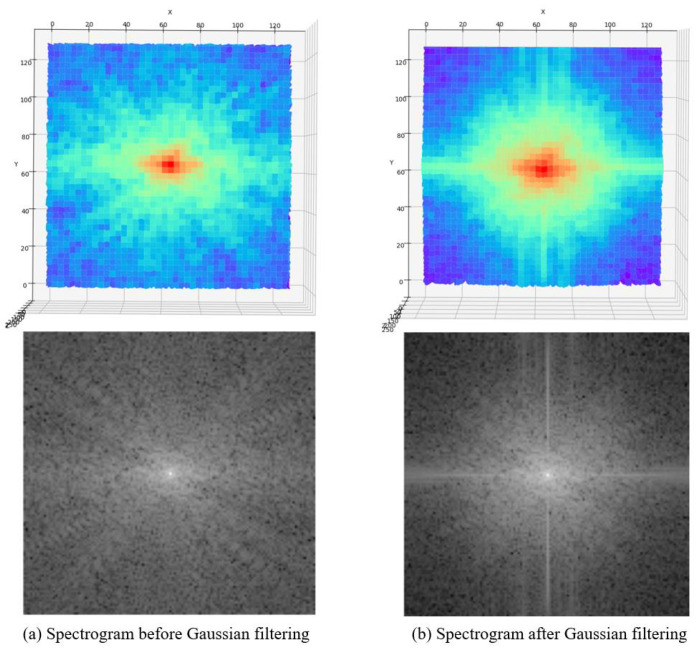
Changes in the spectrogram before and after Gaussian filtering.

Convolutional neural networks are more suitable for early vision processing, which can produce more stable and high-quality results. In addition, our shallow feature extraction network also includes processing low-frequency information of images. The low-frequency information of the image often refers to the continuous gradient area in the picture. That is, the image content within the edge is the low-frequency information. The image’s low-frequency information is the image’s preliminary information, which contains the rough outline and general appearance. In the high-resolution image reconstruction task, the low-frequency information carried by the low-resolution image (LR) and the super-resolution image (SR) is similar, so it is necessary to extract the low-frequency information. Therefore, in our shallow feature extraction network, we increase the processing of low-frequency information. After a layer of Gaussian filter, the low-frequency information is well preserved, and the high-frequency information is filtered (see Figure 7). There are not many detailed textures in anime images and many color areas are in blocks, so the low-frequency information occupies a considerable part. Better utilization of low-frequency information is helpful for subsequent image reconstruction work.

**Figure 8:**
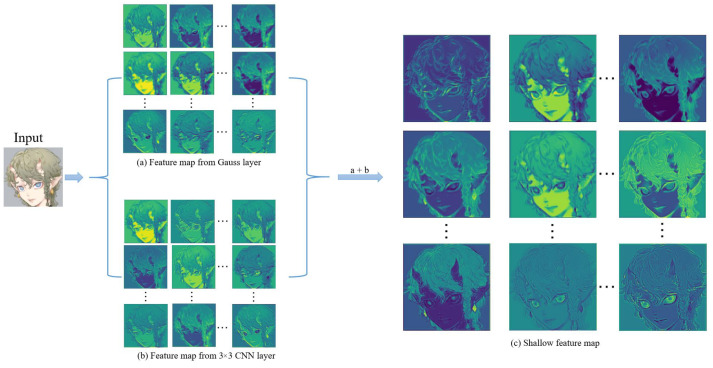
Feature maps extracted by different parts of the shallow feature extraction network.

A low-resolution image (LR) is input as $I_{LR}\in R^{H\times W\times C}$(H,W,C represent the height, width, and channel of the LR image, respectively). We use a single 3 × 3 convolutional layer $H_{LF1}\left(\right)$ of the first part to take its extracted features $F_{L}\in R^{H\times W\times C}$ (see Figure 8):

(1) $$F_{L}=H_{LF1}\left(I_{LR}\right)$$

In order to better preserve the low-frequency information of the image, we chose to use a low-pass filter layer $H_{LP}\left(\right)$. The feature $F_{L}$ we extracted earlier will be input to this layer.

(2) $$F_{Low}=H_{LP}\left(F_{L}\right)$$

In order to better obtain high-frequency features of images, we hope that the input of our deep feature extraction network contains more texture detail feature changes. In Alexnet [36], it is mentioned that the most original texture detail feature changes of the image should be captured with large convolution kernels, such as 7 × 7, 11 × 11 convolution kernels, to extract features. Large-sized convolution kernels can bring a larger receptive field but also introduce a larger amount of computation and more parameters, so we used three 3 × 3 convolution kernels instead of 7 × 7 convolution kernels. We added three convolutional layers $H_{LF3}\left(\right)$ with kernel size 3 after both $H_{LP}\left(\right)$ and $H_{LF1}\left(\right)$.

**Figure 9:**
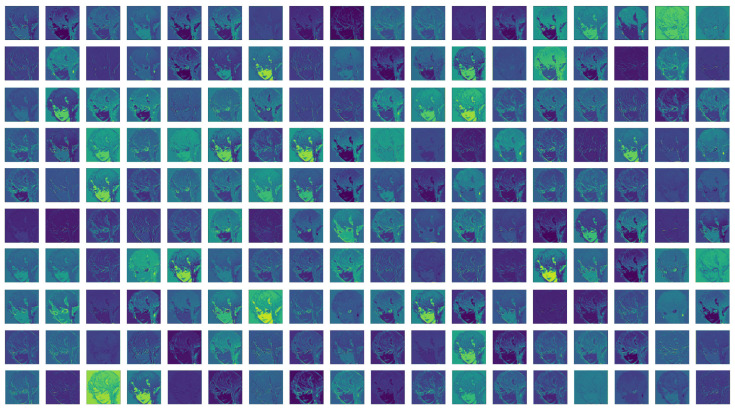
Shallow feature map.

Then, we continued to extract features (feature map shown in Figure 9), which is mainly used to further extract the features of the low-resolution image (LR) and use them as the input $F_{Lin}\in R^{H\times W\times C}$ (where *C* is the feature channel number) of the deep feature extraction network.

(3) $$F_{Lin}=H_{LF3}\left(F_{L}\right)+H_{LF3}\left(F_{Lowf}\right)$$

### 2.2. Deep Feature Extraction Network

Deep features are image features extracted by deep networks (based on Swin Transformer). Each pixel contains information about its region or neighboring regions. Deep features are relatively less fine-grained than shallow features but have rich semantic information.

#### 2.2.1. General Definition

**Figure 10:**
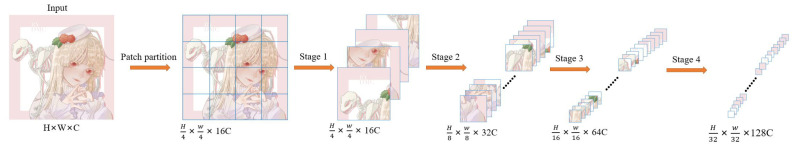
Architecture of a Swin Transformer.

Swin Transformer [27] is a transformer model with a hierarchical design that includes shifted window operations. It splits the image into windows of the same size and computes self-attention only within the windows each time. In order to obtain more global information, Swin Transformer proposes a shifted window operation. Restricting attention computation to a window can introduce the locality of CNN convolution operations on the one hand, and save computation on the other. In addition, the entire model adopts a hierarchical design, including a total of four stages (see Figure 10).

**Figure 11:**
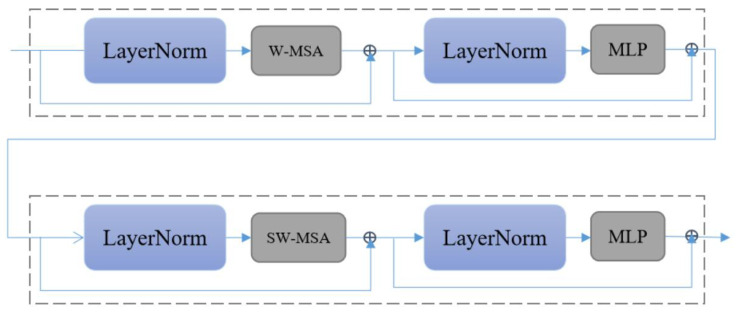
Two successive Swin Transformer layers. W-MSA is window self-attention mechanism. SW-MAS is shifted window self-attention mechanism.

Each stage will reduce the resolution of the input feature map and expand the receptive field layer by layer, such as CNN. The Swin Transformer layer in each Swin Transformer block is an even number, that is, a Swin Transformer layer (STL) without the shifted window and a Swin Transformer layer with the shifted window. Swin Transformer proposes a self-attention model with shifted windows. Through the concatenated window self-attention operation and the shifted window self-attention operation, the Swin Transformer can obtain a near-global attention capability while reducing the amount of computation from the square relationship of the image size to a linear relationship, which significantly reduces the amount of computation (i.e., improved model inference speed). The Swin Transformer layer (see Figure 11) is the base layer in the Swin Transformer.

The feature vectors extracted by our shallow network are replicated in triplicate, named Q,K,V. The specific calculation formula is as follows:

(4) $$Attention\left(Q,K,V\right)=softmax\left(\frac{QK^{T}}{\sqrt{d_{k}}}\right)V$$

#### 2.2.2. Swin Transformer Block (SWTB)

**Figure 12:**
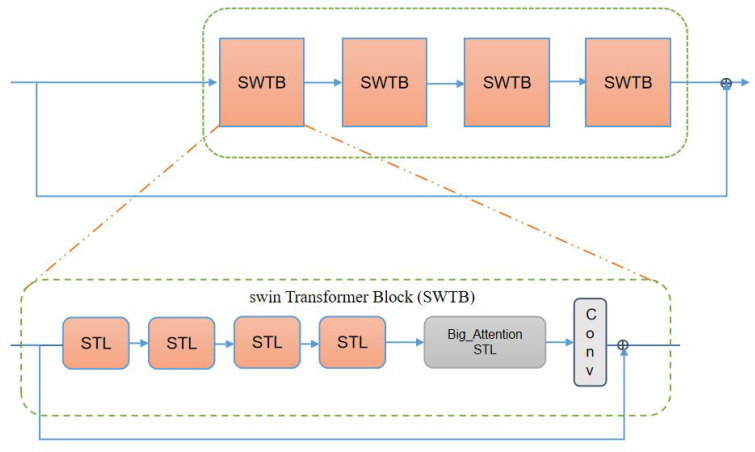
Swin Transformer block (SWTB).

Our deep feature extraction network is mainly based on the Swin Transformer. We removed the patch merging operation to keep our feature map size in a constant state. Our deep feature extraction network is composed of four Swin Transformer blocks (see Figure 12), and each SWTB has four Swin Transformer layers and a big attention layer.

This is the same as the method used in the Swin Transformer; four layers of Swin Transformer alternately use Swin Transformer layer without shifted window and Swin Transformer layer with shifted window. Nevertheless, we removed the parch merging part of the Swin Transformer. This is because the parch merging operation in the Swin Transformer is mainly proposed for classification tasks. Since we choose a post-upsampling network structure, we do not want the feature map to keep shrinking in size during feature extraction. The process of shrinking the feature map will bring about the exponential growth of the feature dimension, which is a big test for the computer’s computing power. In addition, reducing the feature map size requires subsequent upsampling operations. Consecutive upsampling operations may cause checkerboard artifacts because the size of the convolution kernel is not divisible by the stride, although it is possible to perform the interpolation resize operation and then perform the deconvolution operation to avoid this. However, the error introduced by the interpolation will lead to the degradation of the quality of the generated image. Therefore, we choose to keep the feature map size unchanged.

Although the Swin Transformer achieves a certain degree of global attention mechanism through clever shifting, it is limited by the size of the window and cannot obtain a global interaction to the greatest extent. To make up for this defect, the Swin Transformer introduces a hierarchical design. It reduces the feature map size through continuous patch merging operations. Thus, a larger receptive field can be obtained by using a window of a certain size.

**Figure 13:**
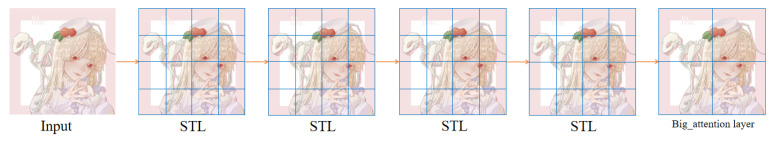
Self-attention window changes in a deep feature extraction network. In the beginning, we divide the image into several small windows. In the last stage, we divide the image into four regions.

The patch merging operation is removed because we want the feature map size to remain the same. This causes our receptive field to become very small. In order to solve this problem, we add a big attention module to each SWTB. We do not increase global awareness by reducing the size of the feature maps. We increase global awareness by expanding the window (see Figure 13).

For example, in the beginning, we calculate the self-attention mechanism in a window of 8 × 8 size. The image features extracted by the shallow feature network are imitated into three copies, *Q*, *K*, and *V*. The weight coefficient is calculated by QK and weighted with *V*. This way, our feature vector *V* contains the position information of different pixels in the 8 × 8 area. If this area is enlarged, the range perceived by the feature vector *V* will be enlarged.

Each SWTB has a 3 × 3 convolutional layer at the end to keep the number of channels unchanged. In addition, we use residual connections in each SWTB. On the one hand, it helps model training. On the other hand, it solves the problem of network degradation.

**Figure 14:**
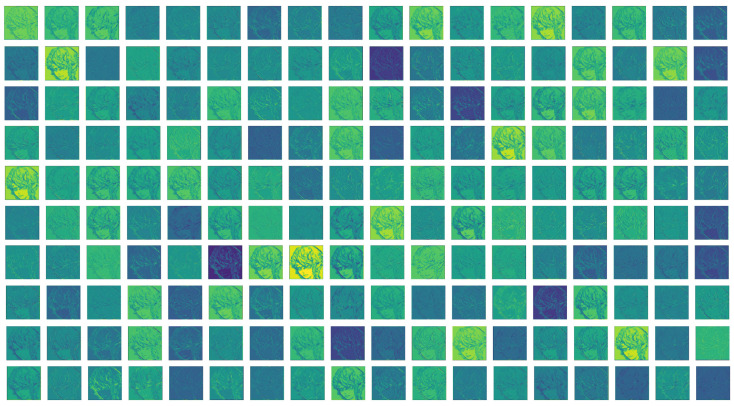
Deep feature map.

The input of the deep feature extraction network is $F_{Lin}$. Through this network, we can extract the feature $F_{High}\in R^{H\times W\times C}$ (see Figure 14).

(5) $$F_{High}=H_{HF}\left(F_{Lin}\right)$$

where $F_{HF}\left(\right)$ is the deep feature extraction network, which contains four SWTBs. Each SWTB is connected in series to gradually extract features $F_{i}\left(i\in 1,2,3,4\right)$.

(6) $$F_{i}=H_{{SWTB}_{i}}\left(F_{i-1}\right)\left(i\in 2,3,4\right)$$

(7) $$F_{High}=F_{4}$$

### 2.3. Reconstruction Network

Image super-resolution reconstruction based on deep learning requires upsampling the image to obtain the output. Therefore, the image reconstruction network is mainly based on the upsampling technique. Traditional upsampling techniques are mainly based on interpolation. However, these interpolation methods will cause the image to be smooth and blurry. Some even have severe loss of details. This is because the new pixels obtained by the interpolation-based method ultimately depend on the surrounding pixel information, which will inevitably lead to the above problems. Another standard method is to use transposed convolution. The transposed convolution first fills the low-resolution image with 0 and then obtains the output through the convolution to obtain the enlarged size image. The neural network can learn the parameters of the transposed convolution, which is widely used in the super-resolution task. Nevertheless, this method can easily lead to uneven overlap on different axes, which leads to a “checkerboard effect” that impairs the reconstruction performance.

**Figure 15:**
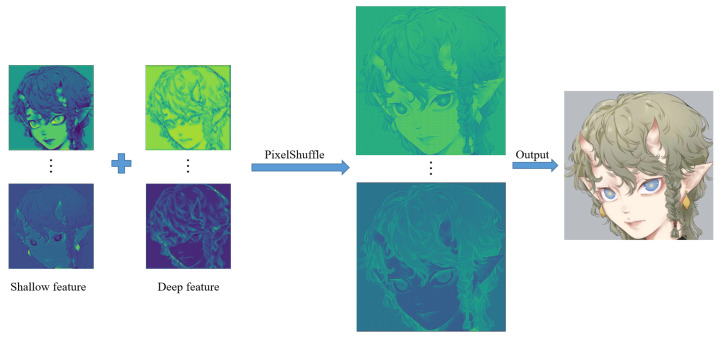
An upsampling example of reconstruction work.

Moreover, sub-pixel convolution [18] provides us with a new solution. The core idea of the sub-pixel is to obtain a large number of feature map channels through the convolution layer of the network and then arrange and tile these feature channels to obtain an image of a predetermined size. Therefore, sub-pixel convolution can utilize more contextual information to restore realistic details. It captures global features nicely. We combine the previous shallow and deep feature information in the image reconstruction network (see Figure 15). Suppose the final image is $I_{SR}$, and $H_{rec}$ is the reconstruction network.

(8) $$I_{SR}=H_{rec}\left(H_{LF3}\left(F_{L}\right)+F_{High}\right)$$

### 2.4. Loss Function

In order to reduce the pixel difference between the output image of the reconstructed network and the accurate super-resolution image information, the similarity at the pixel level is improved by reducing the $L_{1}$ distance between the generated image and the actual image of the training set. The $L_{1}$ loss function can be expressed as

(9) $$L_{1}=\|I_{rec}-I_{HR}{\|}_{1}$$

### 2.5. Network Parameter Settings

The ordinary convolution layer in the shallow feature network uses a 3 × 3 convolution kernel, the stride is 1, and the padding is 1. The Gaussian convolution layer uses a 5 × 5 convolution kernel, the stride is 1, and the padding is 2. The network parameters of each SWTB in the deep feature extraction network are as follows: the window size of the first four layers of STL is set to 8, and the window size of the last layer of STL is set to a quarter of the input image size. The ratio of MLP (multilayer perceptron) is 2, and we set the number of heads in multi-head attention to 6. We set the number of channels of shallow feature extraction network and deep feature extraction network to 180, and the total number of parameters of our network is 7.9 M.

## 3. Dataset

### 3.1. Constitution of the New Anime Dataset

Three major factors that affect the development of deep learning are algorithms, data, and computing power. All three are significant to any research study. Data are the foundation, and any research is inseparable from data support. The corresponding research work cannot be carried out without a dataset. Currently, there are very few datasets in the field of anime image super-resolution. The most common one is Manga109 [33], but the number of samples it provides is only 109. Therefore, limited samples are not conducive to the training of neural network models. The large-scale dataset Danbooru [34] provides larger-scale samples, but due to the random collection of datasets, many pictures contain many wrong pictures. This also makes it unsuitable for our study, so we designed our own dataset.

We constructed our dataset by collecting anime images from two popular websites (http://seeprettyface.com [37] and https://www.pixiv.net [38], accessed on 18 October 2022). There is also some anime images from the artist Miss Dai that we use for display in this paper [3]. Seeprettyface provides a dataset of 140,000 anime faces. These anime faces cover almost the vast majority of anime works. That ensures the diversity of data to a certain extent. In addition, these data were preprocessed to ensure the same size. Therefore, we selected 10,000 anime face images from Seeprettyface.

Pixiv is a virtual community site in a social networking service centered on illustrations, comics and novels, and art. The main content of the website is original drawings submitted by users. The source of the theme of the work is generally the fan art of Japanese animation, Japanese manga, video games, or pure original art. In addition, anime works published on Pixiv tend to be of high resolution, which aligns with our research purpose. Pixiv also contains a wide variety of anime works. We scraped 10,000 anime images from Pixiv as our data. Finally, we set up our datasets based on these data for training (8000 images), validation (500 images), and testing (275 images).

#### 3.1.1. Training Datasets

The training dataset consists of 8000 anime images selected from Seeprettyface (10,000 images) and Pixiv (10,000 images). To ensure the diversity of the data, we randomly selected 8000 anime images. The training dataset mainly comprises anime faces, full-body, and half-body images.

**Figure 16:**
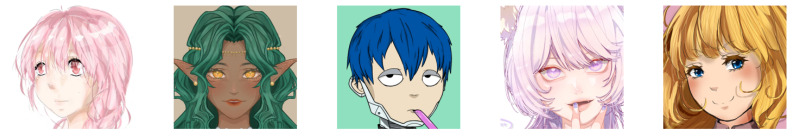
Example images from the anime face dataset (from Seeprettyface and Pixiv). HR is 512 × 512, LR are 64 × 64, 128 × 128, 256 × 256. The images are adopted from [37,38].

Some samples of anime face images are shown in Figure 16. The high-resolution image size is 512 × 512, and low-resolution images of different resolutions are obtained by downsampling.

**Figure 17:**
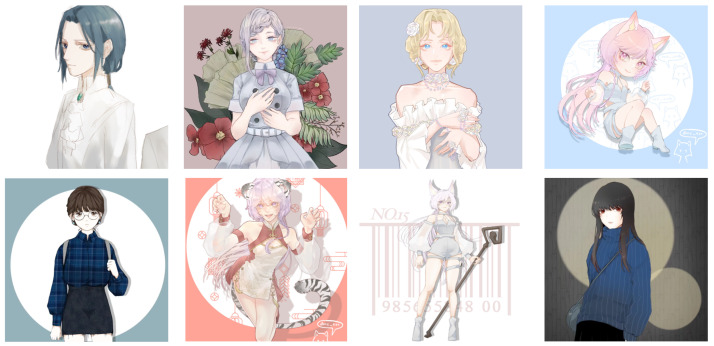
Example images from the anime character image data (full-body and half-body; from Pixiv). HR is 512 × 512, LR are 64 × 64, 128 × 128, 256 × 256. The images are adopted from [3].

Some samples of anime characters images are shown in Figure 17. The high-resolution image size is 512 × 512, and low-resolution images of different resolutions are obtained by downsampling.

#### 3.1.2. Validation Datasets

To ensure the diversity of data, we randomly selected 500 anime images from Seeprettyface (10,000 images) and Pixiv (10,000 images), except for training and testing data.

#### 3.1.3. Test Datasets

Previous super-resolution methods’ performances were evaluated on several public test sets. The most widely used standard test datasets were Set5 [33], Set14 [39], BSD100 [40], and Urban100 [41]. These datasets consist of pictures of life in different scenarios. According to the design characteristics of these datasets, we designed several datasets suitable for testing our methods. This work considers three test datasets (i.e., a total of 275 images) as follows: (a) the AnimeFace180 dataset, containing 180 anime face images, (b) the AnimeCharacter12 dataset, containing 12 anime characters, and (c) the Multi-level anime83 dataset, containing 83 more types of anime images (characters, animals, words, buildings).

**(a) AnimeFace180** AnimeFace180 [37] consists of the faces of 180 utterly different anime characters. The purpose of the AnimeFace180 test dataset is to measure the model’s ability to reconstruct faces. The resolution of the original image is 512 × 512. By downsampling, we obtain low-resolution images of 64 × 64, 128 × 128, and 256 × 256 sizes. In addition, since there is a copyright problem with the image of AnimeFace180, the sample image cannot be shown in this paper.

**Figure 18:**
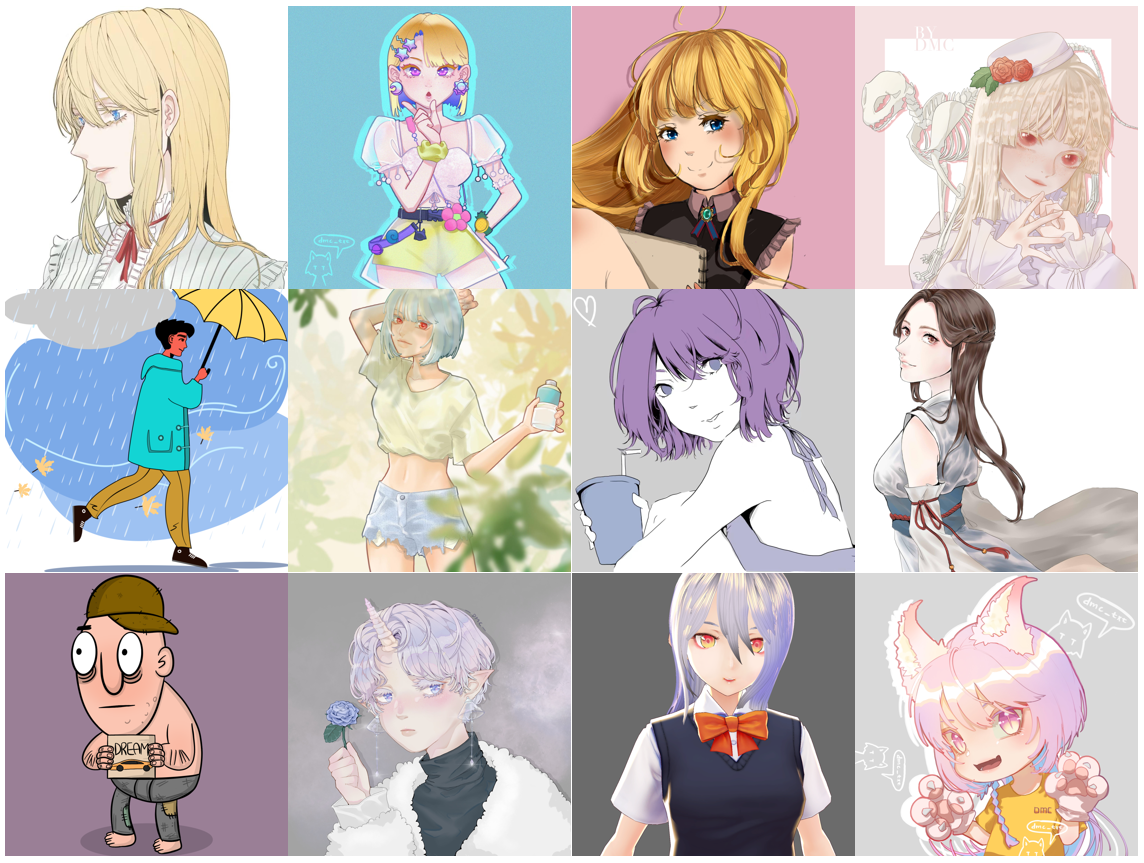
Example images from the AnimeCharacter12 dataset. HR is 1024 × 1024, LR are 128 × 128, 256 × 256, 512 × 512. The images are adopted from [3].

**(b) AnimeCharacter12** AnimeCharacter12 [3] (Figure 18) consists of 12 utterly different anime character images. The original intention of AnimeCharacter is to test the overall super-resolution effect of the model on anime characters. The resolution of the original image is 1024 × 1024. By downsampling, we obtain low-resolution images of 128 × 128, 256 × 256, and 512 × 512 sizes.

**Figure 19:**
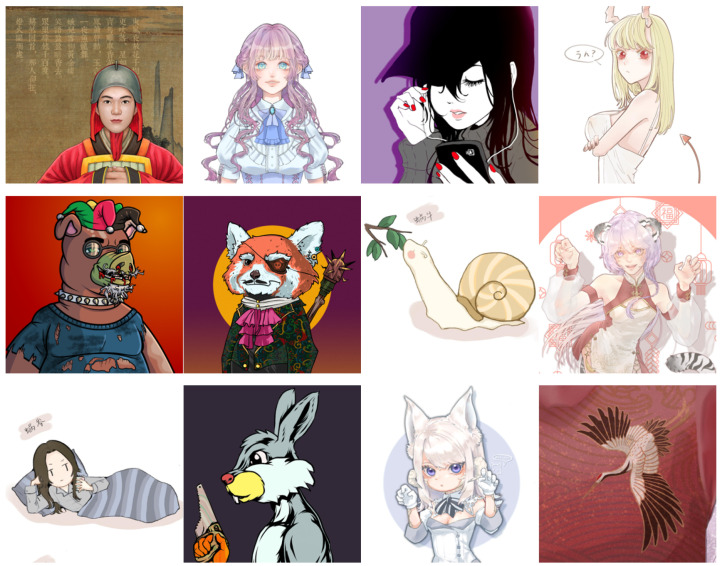
Example images from the Multi-level anime83 dataset. HR is 1024 × 1024, LR are 128 × 128, 256 × 256, 512 × 512. The images are adopted from [3].

**(c) Multi-level anime83.** The dataset of the Multi-level anime83 [3] (see Figure 19) consists of 83 anime images. The purpose of the Multi-level anime83 dataset is to test the robustness of the model because Multi-level anime83 contains image types that are not in the training set, such as animals, Chinese characters, and buildings. The resolution of the original image is 1024 × 1024. By downsampling, we obtain low-resolution images of 128 × 128, 256 × 256, and 512 × 512 sizes.

## 4. Experimental Results

### 4.1. Environment Settings

The configuration of the experimental environment is RTX3080, 16 GB memory. Cuda version is 11.3. We used version Cudnn 8.2.1 to speed up the training. The experimental environment is configured as anaconda3, torch1.10, and python3.8. The training visualization uses the wandb third-party library [42]. In addition to the conventional libraries, toolkits such as torchvision [43], OpenCV [44], and timm [45] need to be installed in Python. The optimization of the model in the experiment adopts the Adam algorithm. Although the model has a high degree of complexity, the probability of underfitting is small. Nevertheless, we do not add a regularization parameter to the loss function to reduce the possibility of underfitting. In addition, we avoid the overfitting problem by dropout operation. We set the initial learning rate to 7 × 10${}^{5}$ and dynamically adjust the learning rate over the entire training. Reducing the learning rate at certain moments helps the model train faster. After several trials and errors, we found that the learning rate set at half leads to a relevant result. We identify countermeasures for learning rate decay. The learning rate is large at the beginning and decreases appropriately when the epoch reaches a certain level. The learning rate is decreased by half after the 100th epoch. We decrease the learning rate at [100, 160, 200, 240, 280, 320, 400] epochs. For example, the initial learning rate was 7 × 10${}^{5}$. Later, the learning rate was reduced to 3.5 × 10${}^{5}$ at the 100th epoch.

### 4.2. Image Quality Assessment

Two commonly used indicators to quantitatively evaluate the quality of super-resolution images are peak signal-to-noise ratio (PSNR) and structure similarity (SSIM).

MSE represents the mean square error of the reconstruction image *X* and the original image *Y*. *H* and *W* are the height and width of the reconstruction image and original image. *i* and *j* are pixel index. The larger the PSNR value is, the better the reconstructed image quality is, and its unit is dB.

(10) $$MSE=\frac{1}{H\times W}\sum_{i=1}^{H}\sum_{j=1}^{W}{\left(X(i,j)-Y(i,j)\right)}^{2}$$

(11) $$PSNR=10\log_{10}\left(\frac{{\left(2^{n}-1\right)}^{2}}{MSE}\right)$$

Structural similarity is a measure of the similarity between two images. Given two images *X* and *Y*, the structural similarity of the two images can be calculated according to Equation (10). $\mu_{X}$ is the average of *X*. $\mu_{Y}$ is the average of *Y*. $\sigma_{XY}$ is the covariance of *X* and *Y*. $c_{1}$ and $c_{2}$ are constants. The result of SSIM is between 0 and 1. The closer the value of SSIM is to 1, the more similar the structure between the reconstructed image and the reference image is. On the contrary, it means that the two structures are not similar.

(12) $$SSIM\left(X,Y\right)=\frac{\left(2\mu_{X}\mu_{Y}+c_{1}\right)\left(2\sigma_{XY}+c_{2}\right)}{\left({\mu_{X}}^{2}+{\mu_{Y}}^{2}+c_{1}\right)\left({\mu_{X}}^{2}+{\mu_{Y}}^{2}+c_{2}\right)}$$

### 4.3. Results

#### 4.3.1. Results on Anime Face and Characters

In order to assess the effect of our model’s performance on the super-resolution of anime images, we selected 180 anime face images (AnimeFace180) and 83 anime character (Multi-level anime83) images from the test dataset for testing. The image features of AnimeCharacter12 are included in the image of Multi-level anime83, so they are not used in this test.

**Figure 20:**
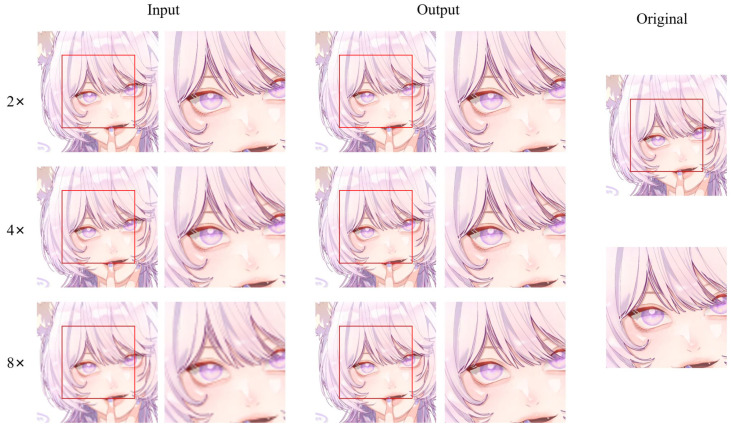
An example of visual result of an anime face on test dataset (AnimeFace180). To show the details of the generated anime image, we zoomed in on the anime face. The red box area is the area we intercepted and enlarged—visual result on the anime face.

An example of different super-resolution tasks (2×, double super-resolution; 4×, quadruple super-resolution; 8×, octal super-resolution) is shown in Figure 20. The example comes from the test dataset consisting of 180 anime face images. Following Figure 20, we can conclude that in 2× and 4× tasks, the anime image’s sharpness significantly improves. The jagged lines in some of the original low-resolution images are smoothed out. The difference between the generated image and the original high-resolution image is minimal. In the 8× task, the jagged lines in the original low-resolution image were improved, and the overall quality of the image was also improved compared to before.

**Figure 21:**
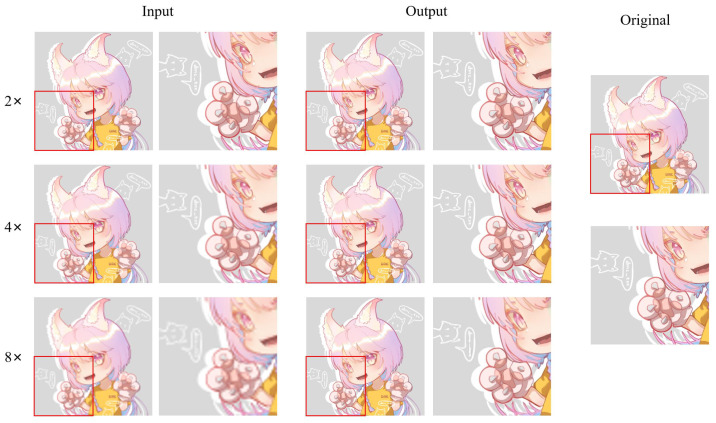
An example of visual result of an anime character on test dataset (Multi-level anime83). The red frame area is the hand details.

In addition to anime face images, there are also many half-body and full-body images in anime works. Therefore, we selected 83 anime character images, including half-body and full-body images, from the test dataset to assess the model’s performance. Figure 21 shows an example of different super-resolution tasks. We zoomed in on parts of the image to show details more clearly. In the 2× and 4× tasks, the details of hair end and hand nails in the anime images generated by our model are significantly improved. In addition, the lines across the character are smoother than in the original low-resolution image. In the 8× mission, the sharpness of the characters is also greatly improved. The test results of anime faces and characters show that our model has a good effect on improving the resolution of low-resolution anime images. Furthermore, Table 1 demonstrates the maximum and average values of PSNR and SSIM for the super-resolution tasks (2×, 4×, 8×) on these two test datasets. The standard value of PSNR in lossy image and video compression is said to be 30–50 dB, but the average value is over 30 dB even at 8×. It is said that quality degradation is visibly noticeable when the SSIM is 0.90 or less, but the average value is above this value up to 4× task.

#### 4.3.2. Ablation Studies

In this section, we ablate essential design choices in our model. For the ablation study, we trained our model on the part of the training dataset and tested it on the Animeface180 and Multi-level anime83 test datasets. The model that removes the shallow feature extraction network is called AISR-XS. The model that removes the enlargement window operation (big attention layer) is called AISR-XW. The model that removes both operations is called AISR-XSW. Finally, the model with both operations is called AISR-O.

We take AISR-XSW, removing the shallow feature extraction network and expanding the window mechanism as the baseline. As shown in Table 2, when dealing with low-resolution images of 128 × 128, using the shallow feature extraction network alone improves the PSNR indicator by 0.05 dB, and using the expend window mechanism improves the PSNR indicator by 1.03 dB. When both are used, the PSNR index is improved by 1.47 dB. The SSIM indicator also improves from the initial stage. When dealing with 256 × 256 low-resolution images, using the shallow feature extraction network improves the PSNR index by 0.75 dB, and using the expend window mechanism improves the PSNR index by 0.84 dB. When both are used, the PSNR index is improved by 1.06 dB. The SSIM indicator also improves from the initial stage. From Table 2, we confirm that each of our modules contributes to improving the final result.

#### 4.3.3. Comparison against the State-of-the-Art Methods

In order to reflect the different performances among different models, the model proposed in this paper is compared with the classic or state-of-the-art methods.

**Bicubic:** For the classic super-resolution, we choose the Bicubic method. Bicubic interpolation [4] is a more complex interpolation method. The algorithm uses the gray value of 16 points around the point to be sampled for cubic interpolation, which takes into account the gray value of the four directly adjacent points and the effect of the gray value change rate between adjacent points. Three operations can approach the upscaling effect of high-resolution images; it was widely used in super-resolution tasks in the early days. The traditional interpolation stretching and enlarging methods can hardly avoid problems such as aliasing, blurred images, and missing data. We use cubic interpolation in OpenCV to upscale the anime image by 2×, 4×, 8×.

**Waifu2x:** Waifu2x [32] uses a trained deep convolutional neural network for image enlargement, which solves the shortcomings of other enlargement methods, such as reduced line sharpness and poor color block purity which often occur when enlarging animation-style pictures. It outperforms other magnification methods. We use Vgg7 to extract features and then use upconv7 to upsample to obtain the final 2× result. The result of 4× is performing two 2× operations. The result of 8× is four 2× operations.

**Real-ESRGAN:** In addition, we also choose a GAN-based anime image super-resolution method. Usually, an actual image may go through various processes, such as camera blur, sensor noise, and image compression, that make the image blurred and degraded. Therefore, Real-ESRGAN [23] extends the first-order degradation model to a higher-order degradation model. A filter is then set up to simulate ringing and overshoot artifacts. The result is that this model is able to restore images more. Moreover, Real-ESRGAN was trained separately for anime images and achieved good visual effects. The generator we used is a deep network with several residual-in-residual dense blocks. The discriminator we used is a U-Net with spectral normalization.

**SwinIR:** Since the transformer-based method is not currently applied to anime image super-resolution, we choose the current state-of-the-art method for comparison. SwinIR [28] is based on Swin Transformer. SwinIR has a strong global perception ability to improve the overall quality of the image. For SwinIR, RSTB numbers and STL numbers are 6 and 6.

Comparative experiments were performed based on previously designed datasets. Additionally, quantitative comparisons were made on the result values (PSNR, SSIM). A visual comparison was also performed for graphic illustration.

(1) Comparison on AnimeFace180

**Figure 22:**
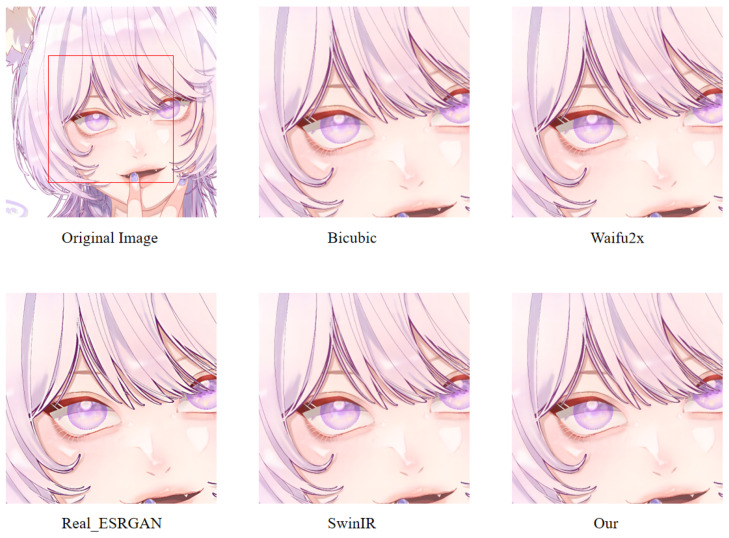
A result of an example image from the Animeface180 test dataset for different super-resolution methods. Facial detail comparison at 2× super-resolution task.

**Figure 23:**
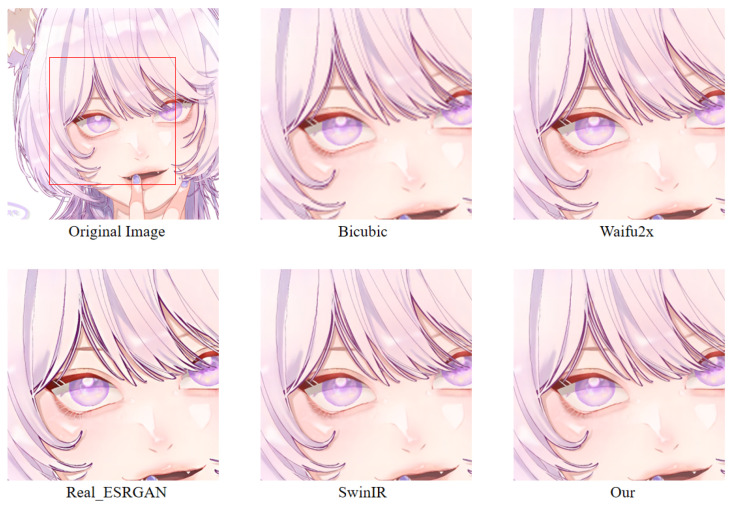
A result of an example image from the Animeface180 test dataset for different super-resolution methods. Facial detail comparison at 4× super-resolution task.

**Figure 24:**
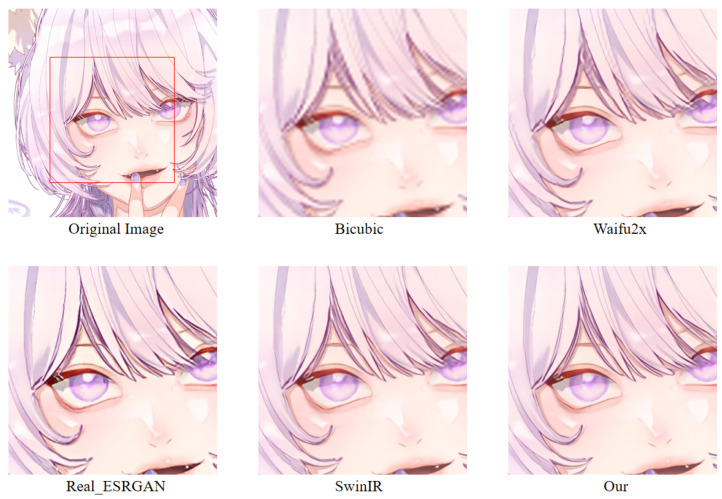
A result of an example image from the Animeface180 test dataset for different super-resolution methods. Facial detail comparison at 8× super-resolution task.

In order to more intuitively observe the anime image reconstruction capabilities of different models, we visually compare the reconstructed images as shown in Figure 22, Figure 23 and Figure 24.

We can see the details of different models’ performances in the reconstructed images (see Figure 22, Figure 23 and Figure 24). Bicubic-based methods achieve the worst results. We stated this point before because the interpolation-based method relies too much on the information of the image itself, so the reconstruction effect is often not good. Some ghosting and checkerboard effects appear in images reconstructed by Waifu2x. However, the images generated by Real-ESRGAN reconstruction avoid the occurrence of such ghosting and checkerboard effects. Moreover, if we only measure from a visual point of view, we see that Real-ESRGAN generates images of decent quality.

Nevertheless, since Real-ESRGAN is based on GAN, some hallucinations will appear during anime image reconstruction. It will add some extra information, such as the thick lines at the corners of the eyes. For SwinIR, the overall effect of anime images generated by SwinIR is greatly improved. However, because it removes the patch merging operation, the global perception ability is reduced, and some image details are missing. Finally, the model based on our method reconstructs images with fewer artifacts and avoids the checkerboard effect. Table 3 discloses the assessment of our proposed method against the state-of-the-art methods. The presented experimental results demonstrate that the proposed method achieved the best results, followed by SwinIR. However, as mentioned above, the Bicubic-based method performed the worst. The assessment was performed using the standard PSNR and SSIM metrics on resolution tasks at different magnifications (2×, 4×, 8×).

(2) Comparison on AnimeCharacter12

In Animeface180, we only tested the super-resolution effect of anime face images. However, there are not only a large number of anime face images in anime works, but also many full-body images of anime characters. Therefore, to assess our model’s performance in the overall anime character, we conduct comparisons between different methods on the AnimeCharacter12 test dataset.

**Figure 25:**
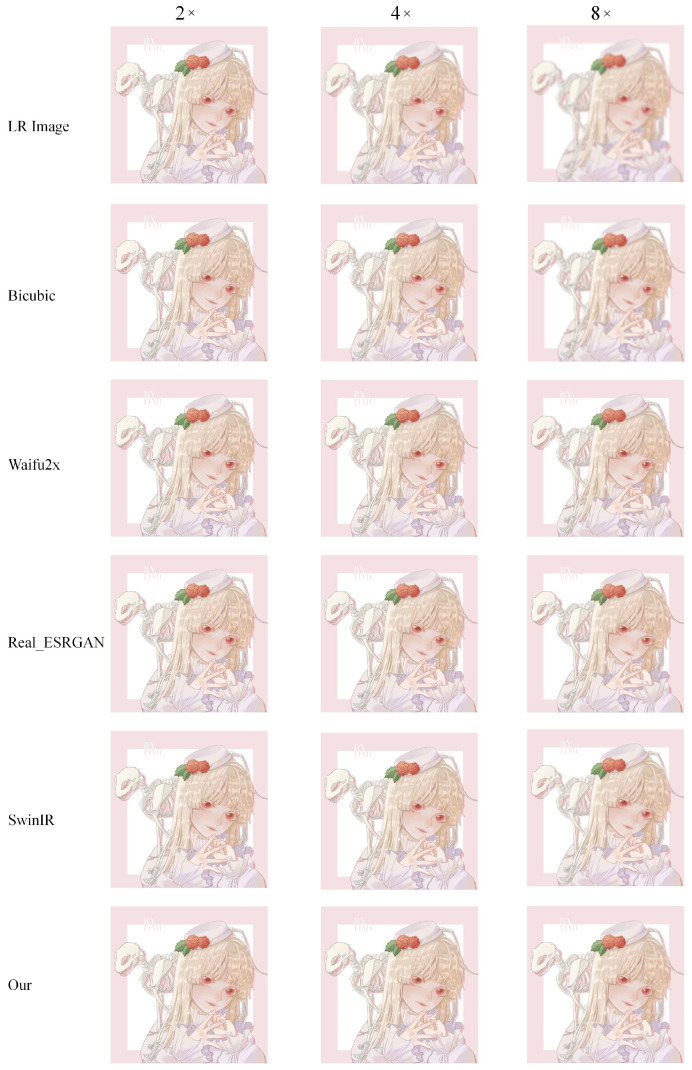
Result of an example image from the AnimeCharacter12 test dataset for different super-resolution methods at 2×, 4×, and 8× super-resolution task.

First, we use an example to visually compare the differences between different methods (see Figure 25).

The super-resolution image obtained by the interpolation method, i.e., Bicubic, which relies on the image’s information, is still very blurry and does not have the effect of improving the image resolution. The results achieved by Waifu2x on 2× missions are still good, with no obvious error messages. However, on 4× and 8× tasks, Waifu2x has some glaringly wrong textures. For example, the color of some areas overflows, and some lines are jagged. This is because Real-ESRGAN mainly focuses on visual perception, resulting in the images generated by it not having high values in evaluation indicators. While visually pleasing to the human viewing experience, there are some color deviations from high-resolution images, and some lines are overly thickened. SwinIR and our method are based on the Swin Transformer, and the images generated by SwinIR and ours are closer to the original high-resolution image. Nevertheless, because we make good use of the rich low-frequency information of the anime image, the color in the enclosed area is closer to the original image. In addition, the increased global awareness also makes our method smoother than SwinIR on some fine lines.

By comparison, our model is better than Waifu2x in detail rendering. Moreover, compared to Real-ESRGAN, we did not have some hallucinations problems. Compared to SwinIR, we are better at the restoration of details. This is thanks to the last large attention window in each layer of our network. Larger attention windows can incorporate more information. Moreover, Table 4 also confirms the performance assessment of our model, which achieved the best results in terms of standard PSNR and SSIM evaluation metrics.

(3) Comparison on Multi-level anime83

Multi-level anime83 contains anime images in different scenes. Therefore, it can well reflect the generalization ability of the model. We verify the generalization ability of different models through Multi-level anime83 (see Table 5).

**Figure 26:**
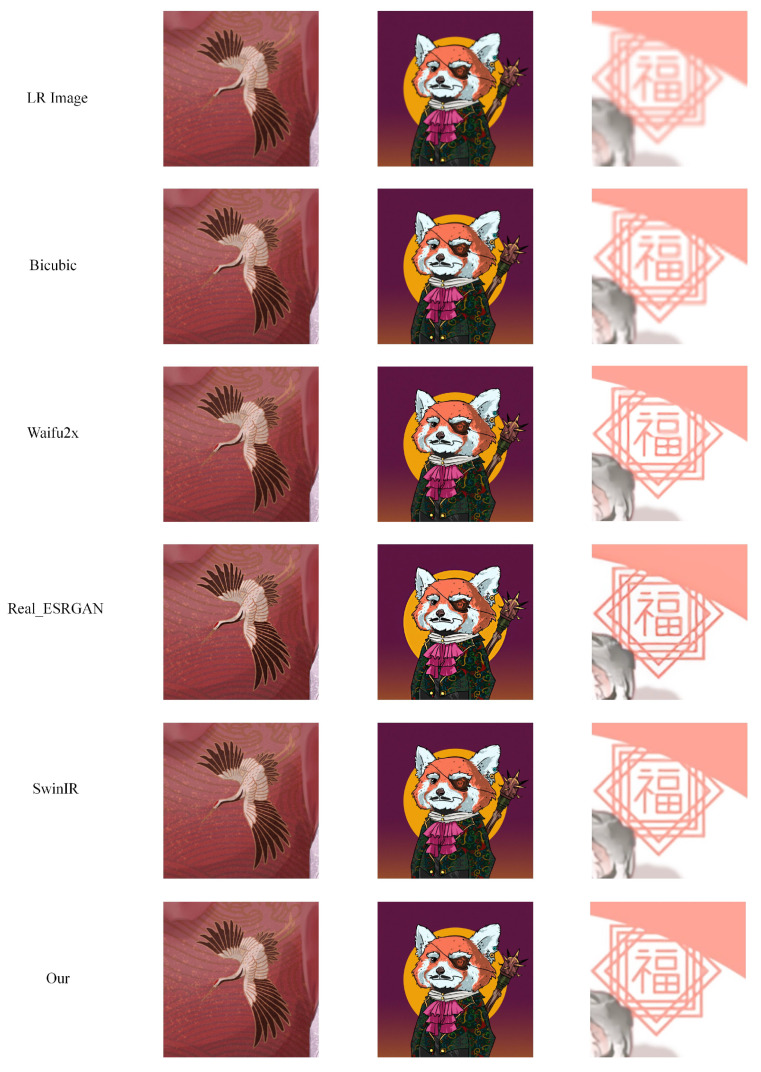
The results of three example images from the Multi-level anime83 test dataset for different super-resolution methods at 4× super-resolution task.

The results of three example images from the Multi-level anime83 test dataset for different super-resolution methods at 4× super-resolution task are shown in Figure 26. Compared with the crane patterns on the clothes of anime characters, the super-resolution images generated by the Bicubic method are still very blurry. The super-resolution image lines generated by Waifu2x are not smooth. The super-resolution images generated by Real-ESRGAN, SwinIR, and ours are not very different from a visual point of view. The same situation occurs in the second super-resolution task on animal characters. However, we can find that the lines generated by Real-ESRGAN are always slightly thicker. The gap between the three can be observed in the third anime picture with Chinese characters. Real-ESRGAN has obvious errors in some strokes. Compared with our generated Chinese characters, the Chinese characters generated by SwinIR have poor regularity and insufficient color uniformity.

Following Figure 26 and Table 5, we can confirm that the proposed model achieves the best results under the 2×, 4×, and 8× tasks, followed by SwinIR. Our model can perform super-resolution tasks well in different application scenarios (anime faces, anime characters, anime buildings, anime animals, etc.).

(4) The runtime comparison with different methods

We conducted runtime tests on three test datasets to verify the computational speed of different methods. AnimeFace180 contains three sizes of anime images: 64 × 64, 128 × 128, and 256 × 256. These sizes correspond to 8×, 4×, and 2× anime image super-resolution tasks. Both AnimeCharacter12 and Multi-level anime83 also contain three sizes of anime images: 128 × 128, 256 × 256, and 512 × 512. These sizes correspond to 8×, 4×, and 2× image super-resolution tasks. The tests were performed on the computer, with CPU-i7-11800H and GPU-3080Ti, respectively.

As shown in Table 6, our model does not have an advantage in runtime testing. As the core of the transformer model, the self-attention mechanism not only endows the transformer with powerful modeling capabilities but also brings a series of computing and memory problems to the transformer model. In computer vision, the self-attention mechanism is based on each pixel as a token, resulting in a longer computing time for the transformer-based model. Our priority in designing the model is the quality of the super-resolution images. Therefore, we disregard memory and computational overhead to obtain better super-resolution results.

### 4.4. Discussion

#### 4.4.1. Resolution of Blur and Checkerboard Artifacts

The main reason for image blur is the lack of detailed image features. The conventional CNN model requires a deep network to obtain a larger receptive field and capture more detailed features. However, deep networks often face the problem of being difficult to train and are underequipped. Therefore, most CNN-based models control the number of network layers within a specific range, reducing the receptive field. This is similar to how the human eye looks at a part of the image simultaneously and then restores the entire image. This challenging task is why the CNN-based model has ambiguous results.

In the proposed method, we obtain local information through the self-attention mechanism of the Swin Transformer and then use the moving window of the Swin Transformer to obtain significant global information. We incorporate more extensive layers of attention mechanisms to capture more global information. The experimental results also demonstrated that adding a more extensive attention layer can help improve the overall effect.

The size of the output image, which is extracted with features through a convolutional neural network (CNN), will tend to be smaller. Therefore, sometimes, we need to restore the image to its original size for further computation. Expanding the image size to realize resolution enhancement is called upsampling. Most of them will use transposed convolution (TC) to complete this process. However, when the kernel size in TC is not divisible by stride, there will be a checkerboard effect. Even if it can be divisible by stride, there will be a checkerboard effect once the learning is uneven. Based on the Swin Transformer, the size of the output image extracted through our network will not change. Therefore, we do not need to restore the image to its original size, avoiding the checkerboard artifacts problem.

#### 4.4.2. Resolution of Ignore Details

To our knowledge, most super-resolution network models focus on deep feature extraction, as the deep features are closer to the final output. Moreover, these models have good performance in extracting deep features. However, shallow features are also important and cannot be ignored. Making better use of shallow features can add more details to image generation. Unlike SwinIR and SMIR, which only use one layer of CNN to extract shallow features, our model adds more low-frequency and texture variation information for shallow feature extraction. With the help of this information, the proposed work achieves some more precise results in the details.

#### 4.4.3. Work Limitations

**(a)**  **The window expansion mechanism is subject to computer computing power**

In our deep feature extraction network, the size of our window expansion is chosen to be a quarter of the input image. Due to our video memory constraints, this is the maximum size we can expand. However, if the memory conditions allow, the wider window will extract more global information.

**(b)**  **The enhancement of low-frequency information cannot adapt to tasks of different scales**

Under different scale tasks, the importance of low-frequency information is different. Especially under the super-resolution of 8×, the low-frequency information of high-resolution images and low-resolution images is quite different. Our method may introduce some wrong information, which leads to some errors in the final result.

**(c)**  **Use of artificially generated low-resolution image**

Real lower-resolution images should be used for further assessment. If we use the collected actual anime data to train our model instead of artificial data, it can be better applied to the animation field. In addition, the real low-resolution images are also of great help to the performance of our test model.

**(d)**  **Longer runtime**

Our model requires higher computational overhead, which results in a longer runtime. The super-resolution task of large-scale images cannot be completed in a short time.

## 5. Concluding Remarks and Future Work

Most of the current anime image super-resolution tasks were accomplished using conventional CNNs, and the use of contemporary transformers that perform equally well in vision tasks has gained significant attention. Transformers have excellent long-distance modeling properties. This is significant in the domain of deep learning. In addition, deep learning models only focus on building deep networks to extract deep feature information while ignoring the importance of shallow feature information. The shallow feature information incorporates a large amount of low-frequency information, and this low-frequency information can well describe the overall structure of the anime image.

Therefore, in response to these problems, we introduced a Swin-Transformer-based anime image super-resolution reconstruction method. Two different neural networks were employed to extract the image’s shallow feature information and deep feature information. This study aimed to propose a neural network that adopts a post-upsampling architecture to reduce the model parameters. Such an architecture allows our model to focus only on feature extraction for low-resolution images. In the final image reconstruction module, we fuse shallow features containing much low-frequency information followed by deep features containing much high-frequency information and reconstruct a high-resolution image through upsampling. In order to verify the effectiveness of the method proposed in this paper, we constituted three datasets, tested and verified each dataset, and performed data analysis and visualization against the popular state-of-the-art methods. It resulted in numerically and graphically verified, robust reconstruction of high-resolution images. The appropriate metric for image quality assessment was indicated based on the evaluation of different structural similarities and peak signal-to-noise ratio, respectively. Assessments of the proposed method confirmed the robustness of the data scenarios: (a) AnimeFace180, (b) AnimeCharacter12, and (c) Multi-level anime83.

For future work, we found through investigation that most anime super-resolution models are equipped with the corresponding user interface. Most people have no prior programming knowledge, so they cannot use the relevant code directly. A user interface can make user operation comfortable, simple, and accessible. Under the 8× task, a low-resolution image needs to be enlarged by a factor of eight, which means that a low-resolution image is only 1/64 of a high-resolution image. Therefore, the low-frequency features extracted by the shallow network will contain some wrong information, resulting in the degradation of image quality. How to effectively fuse low-frequency features is the key to solving this problem. Last, but not least, more and more models are trying to extract features by combining multiple attention mechanisms; for example, HAT [46] was published in May 2022. This paper adopts a parallel approach to combine channel attention and self-attention.

## Figures and Tables

**Table 1:** Results on anime faces (180 images, AnimeFace180) and characters (83 images, Multi-level anime83).

	Anime Faces				Anime Characters			
	Max PSNR	Average PSNR	Max SSIM	Average SSIM	Max PSNR	Average PSNR	Max SSIM	Average SSIM
2×	48.202 dB	40.704 dB	0.996	0.987	42.941 dB	33.598 dB	0.996	0.963
4×	41.251 dB	33.869 dB	0.989	0.959	42.086 dB	32.234 dB	0.992	0.934
8×	33.267 dB	26.554 dB	0.943	0.860	36.024 dB	27.299 dB	0.975	0.866

**Table 2:** Impact of shallow feature extraction part and big attention layer on 4× anime super-resolution.

Methods	Size	Params	Runtime (Image/s)	PSNR	SSIM
AISR-XS	128	7.21 M	5.31	33.424 dB	0.951
AISR-XW	128	6.94 M	5.68	33.246 dB	0.954
AISR-XSW	128	6.07 M	6.06	32.390 dB	0.947
AISR-O	128	8.37 M	4.59	33.869 dB	0.959
AISR-XS	256	7.21 M	0.98	31.952 dB	0.936
AISR-XW	256	6.94 M	1.06	31.864 dB	0.937
AISR-XSW	256	6.07 M	1.19	31.112 dB	0.930
AISR-O	256	8.37 M	0.45	32.175 dB	0.940

**Table 3:** Comparison with other super-resolution methods on AnimeFace180 test dataset (180 images). The final result is the average result of 180 test images in the Animeface180 test dataset (best results are indicated with bold). N = 180.

Method	2×		4×		8×	
	PSNR	SSIM	PSNR	SSIM	PSNR	SSIM
Bicubic [4]	28.973 dB	0.933	25.860 dB	0.828	20.548 dB	0.622
Waifu2x [32]	36.166 dB	0.978	30.088 dB	0.930	24.439 dB	0.805
RealESRGAN [23]	29.657 dB	0.983	28.963 dB	0.935	22.987 dB	0.783
SwinIR [28]	39.553 dB	0.985	32.705 dB	0.950	26.016 dB	0.845
Our	**40.704 dB**	**0.987**	**33.869 dB**	**0.959**	**26.554 dB**	**0.860**

**Table 4:** Comparison with other super-resolution methods on the AnimeCharacter12 test dataset (12 images). The final result is the average result of 12 test images in the AnimeCharacter12 test dataset (best results are indicated with bold). N = 12.

Method	2×		4×		8×	
	PSNR	SSIM	PSNR	SSIM	PSNR	SSIM
Bicubic [4]	33.071 dB	0.951	27.693 dB	0.876	24.669 dB	0.806
Waifu2x [32]	38.536 dB	0.972	31.834 dB	0.934	26.974 dB	0.867
RealESRGAN [23]	32.136 dB	0.954	29.612 dB	0.925	25.733 dB	0.852
SwinIR [28]	38.460 dB	0.971	32.467 dB	0.940	27.601 dB	0.878
Our	**39.137 dB**	**0.973**	**33.081 dB**	**0.943**	**27.873 dB**	**0.883**

**Table 5:** Comparison with other super-resolution methods on the Multi-level anime83 test dataset (83 images). The final result is the average result of 83 test images in the Multi-level anime83 test dataset (best results are indicated with bold). N = 83.

Method	2×		4×		8×	
	PSNR	SSIM	PSNR	SSIM	PSNR	SSIM
Bicubic [4]	31.531 dB	0.946	26.328 dB	0.850	23.331 dB	0.759
Waifu2x [32]	37.524 dB	0.975	30.975 dB	0.932	25.878 dB	0.846
RealESRGAN [23]	31.150 dB	0.953	28.394 dB	0.914	24.502 dB	0.819
SwinIR [28]	37.184 dB	0.973	31.439 dB	0.934	26.661 dB	0.859
Our	**37.990 dB**	**0.975**	**32.175 dB**	**0.940**	**27.026 dB**	**0.865**

**Table 6:** The runtime comparison of our proposed method against the state-of-the-art methods. The best results are indicated with bold.

Methods	Test Dataset	Runtime (Images/s) 2×	Runtime (Images/s) 4×	Runtime (Images/s) 8×
Bicubic	AnimeFace180	92.78	104.65	111.11
Waifu2x	AnimeFace180	19.35	18.87	18.15
Real-ESRGAN	AnimeFace180	2.62	2.23	1.38
SwinIR	AnimeFace180	0.95	2.24	8.57
Ours	AnimeFace180	**0.391**	**4.59**	**6.71**
Bicubic	AnimeCharacter12	36.92	40.54	43.01
Waifu2x	AnimeCharacter12	5.73	4.731	4.32
Real-ESRGAN	AnimeCharacter12	0.41	1.52	2.48
SwinIR	AnimeCharacter12	0.34	0.99	1.94
Ours	AnimeCharacter12	**0.13**	**0.56**	**1.45**
Bicubic	Multi-level anime83	31.32	36.08	38.21
Waifu2x	Multi-level anime83	5.69	4.89	5.42
Real-ESRGAN	Multi-level anime83	0.68	1.99	3.54
SwinIR	Multi-level anime83	0.22	1.35	1.69
Ours	Multi-level anime83	**0.17**	**0.45**	**1.05**

## Data Availability

Not applicable.
